# Changes in the Sociodemographic Factors of Tobacco and Alcohol Consumption in Chinese Adolescents from 2004 to 2011

**DOI:** 10.3390/ijerph15061211

**Published:** 2018-06-08

**Authors:** Li Chen, Ruiyi Liu, Marina Pozhidaeva, Jinqiu Xie, Wei Cao, Fan Zhang, Cesar Reis, Manoj Sharma, Yong Zhao

**Affiliations:** 1 School of Public Health and Management, Chongqing Medical University, Chongqing 400016, China; nameclx@foxmail.com (L.C.); lry981118@foxmail.com (R.L.); m.pozhidayeva@gmail.com (M.P.); jinqiu.x@foxmail.com (J.X.); caoweivie@foxmail.com (W.C.); ava11@126.com (F.Z.); 2 Research Center for Medicine and Social Development, Chongqing Medical University, Chongqing 400016, China; 3 The Innovation Center for Social Risk Governance in Health, Chongqing Medical University, Chongqing 400016, China; 4 Preventive Medicine Department, Loma Linda University Medical Center, Loma Linda, CA 92354, USA; cesarreis@hotmail.com; 5 Department of Behavioral and Environmental Health, Jackson State University, Jackson, MS 39213, USA; manoj.sharma@jsums.edu

**Keywords:** sociodemographic factor, tobacco consumption, alcohol consumption, adolescent, China

## Abstract

Finding ways to reduce tobacco and alcohol consumption among adolescents has been a major public health challenge in China. In relation to this issue, the current study evaluated the changes in the sociodemographic factors of tobacco and alcohol consumption among Chinese adolescents who are 12–18 years old. Trends in sociodemographic factors associated with tobacco and alcohol consumption were investigated based on the 2004–2011 China Health and Nutrition Survey data. Questionnaires that extracted data on tobacco and alcohol consumption (i.e., prior experience of smoking cigarettes and drinking alcoholic beverages) were distributed. Additional variables (e.g., age, residence, gender, etc.) were used in the analyses. Firth penalized logistic regression was conducted with drinking and smoking status variables as the dependent variables. Male adolescents were more inclined to smoke in 2004, 2006, 2009, and 2011 (*p* < 0.05 for all). Adolescents aged 15–16 years were more inclined to smoke compared with those aged 12–14 years in 2004, 2006, and 2011 (*p* < 0.05 for all). Among adolescents aged 15–18 years, older ones were more inclined to not smoke in 2004 (odds ratio (OR) = 0.531, 95% confidence interval (CI) 0.343–0.821). Adolescents who did not attend school were more inclined to smoke in 2004, 2006, 2009, and 2011 (*p* < 0.05 for all). Adolescents who were drinkers were more inclined to smoke in 2004, 2006, 2009, and 2011 (*p* < 0.05 for all). Male adolescents were more likely to drink in 2004, 2006, and 2009 (*p* < 0.05 for all). In 2006 and 2009, adolescents aged 15–16 years were more inclined to drink compared with those aged 12–14 years (*p* < 0.05 for all). Among adolescents aged 15–18 years, older ones were less inclined to drink in 2004 (OR = 0.719, 95% CI 0.527–0.980) and 2006 (OR = 0.716, 95% CI 0.527–0.972). Adolescents who smoked were more likely to drink in 2004, 2006, 2009, and 2011 (*p* < 0.05 for all). The prevalence of tobacco and alcohol consumption among adolescents has not changed significantly. The current study identified adolescent high-risk groups for tobacco and alcohol consumption.

## 1. Introduction

Tobacco and alcohol consumption among adolescents have become an enormous public health burden in China. China is the world’s largest tobacco consumer, with more than 300 million smokers or approximately one-third of the total number of smokers in the world. More than half of China’s daily smokers start smoking before the age of 20. A report showed that 80% of young smokers continue to smoke in adulthood [1]. Adolescent smokers have a higher risk of developing cardiovascular diseases and lung cancer in adulthood compared with non-smoking adolescents, especially in their twilight years [2,3]. In 2014, 5.9% of Chinese adolescents smoked combustible cigarettes, whereas only 0.9% of Japanese adolescents did [4]. The worldwide prevalence of adolescents’ combustible cigarette smoking is measured at 10% [5]. Although the smoking rate of Chinese adolescents is lower than that in the whole world, the huge population base makes the problem appear especially serious. According to the China Youth Tobacco Survey, approximately 9.4 million middle school students have tried tobacco products and one-third of them are now tobacco users. Approximately 20% students aged 13–15 have smoked combustible cigarettes at least once, and 5.9% have smoked in the past 30 days [6]. Therefore, the smoking situation of Chinese adolescents is worthy of attention. Globally, alcohol consumption has caused approximately 3.3 million deaths every year (5.9% of all deaths) and 5.1% of global diseases [7]. In 2008–2010, China consumed an average of 6.7 L of alcohol per person annually (15+ years old), higher than the 6.2 L worldwide alcohol consumption per person annually (15+ years old) [7]. The rate of alcohol consumption among Chinese persons over the age of 15 is close to the highest level in the world (the European average is 10.9 L) [7]. Meanwhile, heavy episodic drinking among China’s 15–19-year-old adolescents is around 12.0% [7]. A study among adolescents in six Chinese cities found that more than 50% had drunk alcohol and nearly 40% had drunk alcohol in the past year [8]. Another study reported that the prevalence of drinking among students (9–21 years old) was 7.3%, and 13.2% of the students were reported to have alcohol-related problems [9]. Numerous studies have shown that alcohol consumption is associated with many diseases. Thus, the high prevalence and consumption of alcohol among Chinese adolescents needs intervention.

Adolescents’ early smoking and drinking behaviors are correlated [10]. The earlier adolescents start smoking, the greater the risk of drinking for lifetime [11]. Previous studies have shown that 75% of adolescent smokers continue to smoke as adults [12,13]. Moreover, alcohol consumption increases the risk of using other substances [14]. Studies have shown that alcohol is a “gateway” substance associated with tobacco, marijuana, and prohibited substances [15]. Furthermore, tobacco and alcohol consumption in early adolescence increases the risk of depression and anxiety [16,17]. In addition, drinking behavior combined with smoking can be a cause of certain diseases. For example, smoking does not increase a woman’s risk of breast cancer alone, whereas smoking increases the breast cancer risk of women who drink alcohol [18].

Previous studies have shown that sociodemographic factors and social influences affect adolescents’ tobacco and alcohol consumption. For sociodemographic factors, the risks of tobacco consumption among 13–15-year-old Chinese adolescents have increased with age [6]. A previous study in Wuhan, China revealed that older adolescents were more likely to smoke [19]. A Chinese survey also showed a gender difference in adolescent smokers, among whom males smoked more cigarettes than females [20]. Male adolescents in rural areas are more likely to smoke than those in urban areas [19]. For social influence, a 2010 study revealed that fathers or mothers had a significant impact on adolescent smoking [21]. Among the sociodemographic factors that influence alcohol intake, the negative effects on young adolescents are more serious than those on older adolescents [22]. Gender is related to the alcohol consumption of adolescents, among whom males drink more alcohol than females [23,24,25]. Compared with girls, boys are more expected to have the characteristics associated with excessive drinking, such as aggression [26]. For social influences, a 2015 study in China showed that adolescents who have jobs had no limitation in alcohol consumption [24]. Furthermore, parental attitude was more closely associated with alcohol use in adolescents [27]. In China, tobacco and alcohol play a key role in social events. Tobacco is associated with a humble and respectful attitude, and alcohol represents a traditional culture of hospitality and friendliness [28]. Most Chinese teenagers believe that smoking and drinking are good for making friends and getting along well with others. This point-of-view has promoted smoking and drinking behaviors among Chinese teenagers, as proven by some studies [29,30]. The sociodemographic changes in tobacco and alcohol consumption among Chinese adolescents should be analyzed. Examining such changes can ultimately provide insights for future intervention and enable researchers to examine the public health policies that affect adolescents.

Using data from the China Health and Nutrition Survey (CHNS), we examined the alcohol and tobacco consumption trends in Chinese adolescents (12–18 years old) over a seven-year period (2004–2011). Tobacco consumption was measured by one behavioral factor, namely, “ever smoked cigarettes (combustible cigarettes)” and alcohol consumption was measured by “drink beer or any other alcoholic beverage”. All respondents were asked whether they drank alcoholic beverages, such as beer or any other alcoholic beverage, and whether they had previously smoked cigarettes. Our study objectives are as follows: (1) to investigate the trends in alcohol and tobacco consumption among adolescents; (2) to examine the changes in the sociodemographic factors of alcohol and tobacco consumption; and (3) to investigate the relationship between alcohol and tobacco consumption among Chinese adolescents (12–18 years old).

## 2. Materials and Methods 

### 2.1. Study Sample: CHNS

The CHNS employed a multistage, random cluster process to obtain a sample from nine provinces (Liaoning, Jiangsu, Shandong, Henan, Hubei, Hunan, Guangxi, Guizhou, and Heilongjiang) and three large municipalities (Beijing, Shanghai, and Chongqing, which were added in 2011) [31] . In each province, cities and counties were stratified by income (low, medium, and high), and two cities and four counties were selected by weighted sampling scheme. Four communities (urban/suburban and village/town) in each city/county were randomly selected. In each community, 20 families were randomly selected. In some cases, new households were recruited to replace people who had moved out for various reasons. This comprehensive dataset aims to study China’s health, population, socioeconomic, and nutrition policies. The official website of the CHNS (http://www.cpc.unc.edu/projects/china) provides detailed information on this survey. 

The present study was based on data from the longitudinal CHNS datasets of 2004 and 2011 and included information on 3167 Chinese adolescents (1078 in 2004, 742 in 2006, 612 in 2009, and 735 in 2011). Zhang et al. [31] provided additional information on the CHNS.

### 2.2. Tobacco and Alcohol Consumption

The tobacco consumption outcome measures included “smoked cigarettes” (yes/no). The alcohol consumption status outcome measure included “drink beer or any alcoholic beverage” (yes/no). Adolescents (12–18 years old) were asked to answer the following questions: (1) Have you ever smoked cigarettes (including hand-rolled or device-rolled)? (2) Have you drunk beer or any other alcoholic beverage this year?

### 2.3. Ethical Considerations

As this study is based on the CHNS open database, the authors’ organization does not need to conduct an ethics review.

### 2.4. Covariates

Additional variables, such as age, gender, ethnicity, type of residence, current education status, household registration type, and family type, were used in the analyses. These covariates were included because the questionnaire required information on adolescents between the ages of 12 and 18. Participants were divided into four groups based on their age (12.00–14.99, 15.00–16.99, 17.00–17.99, and 18.00–18.99 years). Previous studies found that 12–14-year-olds had different tobacco prevalence rates than those over 15 years old [5]. Ethnicity was divided into two groups, namely, Han and minority. The participants were divided based on their areas of residence, namely, urban and rural. For education status, the participants were divided into school pupils and those who did not attend school. Household registration type was divided into urban and rural. Family type was divided into four groups, namely, mother lives with adolescent child, mother does not live with adolescent child, father lives with adolescent child, and father does not live with adolescent child.

### 2.5. Statistical Analyses

Additional variables, such as age, gender, ethnicity, type of residence, current education status, household registration type, and family type, were used in the analyses. The participants’ characteristics were summarized by using frequencies and percentages. A chi-square test for trend was performed to determine the smoking and drinking rates among adolescents from 2004 to 2011. Considering the low prevalence rate of drinking and smoking among adolescents, we adopted the more appropriate penalized logistic regression. Penalized logistic regression proposed by Firth [32] was conducted with alcohol consumption and smoking variables as the dependent variables. Previous studies have reported differences in smoking and drinking rates across age groups [5], we adopted two different models for different age groups. A penalized logistic regression model was developed by using age (12.00–14.99, 15.00–16.99, 17.00–17.99, and 18.00–18.99 years old), gender, ethnicity, type of residence, current education status, household registration type, and family type (nuclear family/single-parent family) as independent variables. A penalized logistic regression model was developed by using age (continuous variable), gender, ethnicity, type of residence, current education status, household registration type, and family type as independent variables among adolescents aged 15–18 years old. All tests were two-sided, and *p*-values less than 0.05 were considered statistically significant. All data analyses were performed using statistical software (SAS version 9.4.3; SAS Institute, Cary, NC, USA).

## 3. Results

### 3.1. Characteristics of Sample

A total of 3167 participants from the CHNS data were included in this study, including 1697 males and 1470 females, answered all the questions. The majority of the participants lived in rural areas. A total of 49.1% participants were 12–14 years old, 29.0% were 15–16 years old, 11.2% were 17 years old, and 10.7% were 18 years old. Among the 84.4% of participants who were pupils, 39.8% were from urban households, 85.8% stated that their father lived with the family, and 90.3% stated that their mother lived with the family (Table 1).

A total of 108 participants, which included 99.1% males, 91.7% Han, and 59.3% residents of rural areas, reported that they had previous smoking experience. Among the 76.9% of smokers who were not attending school, 236 participants reported that they had previous drinking experience; these participants included 81.4% males and 92.8% Han; 45.8% drinkers were not attending school (Table 1).

### 3.2. Trends in Prevalence of Alcohol and Tobacco Consumption

Results indicated that 33 (3.06%), 33 (4.45%), 23 (3.76%), and 19 (2.59%) were smokers in 2004, 2006, 2009, and 2011, respectively. In addition, 65 (6.03%), 59 (7.95%), 52 (8.50%), and 60 (8.16%) of participants in 2004, 2006, 2009, and 2011, respectively, reported having previous drinking experiences. No significant statistical difference was observed in the trends of alcohol and tobacco consumption (Table 2).

### 3.3. Penalized Logistic Regression Analysis for Tobacco Consumption among Participants Aged 12–18

Results based on gender showed that female adolescents were less inclined to smoke compared with males in 2004, 2006, 2009, and 2011. Adolescents who were not attending school were more inclined to smoke than adolescents who were attending school in 2004, 2006, 2009, and 2011. Adolescents with previous drinking experience were more inclined to smoke compared with those without previous drinking experiences in 2004, 2006, 2009, and 2011. In 2006 and 2009, adolescents aged 15–16 were more inclined to smoke than those aged 12–14 years. Adolescents aged 18 were less inclined to smoke compared with participants aged 12–14 in 2004 (Table 3).

### 3.4. Penalized Logistic Regression for Alcohol Consumption among Participants Aged 12–18

The results based on gender showed that female adolescents were less inclined to drink compared with males in 2004, 2006, and 2009. In terms of residence, respondents who lived in rural areas were less inclined to drink than those who lived in urban areas in 2004 and 2006. Adolescents aged 15–16 years were more inclined to drink than those aged 12–14 in 2006 and 2011. Respondents aged 18 years were less inclined to drink than those aged 12–14 in 2011. For current education status, participants who were not attending school were more inclined to drink than students in 2004 and 2009. Adolescents in rural households were more inclined to drink. In 2004, 2006, 2009, and 2011, adolescents who had smoked were more inclined to drink than those without smoking experience (Table 4).

### 3.5. Penalized Logistic Regression for Tobacco Consumption among Participants Aged 15–18 

Male adolescents were more likely to smoke in 2004, 2006 and 2009. Participants who were not in school were more likely to smoke in 2004, 2006, 2009, and 2011. Participants with previous drinking experience were more inclined to smoke in 2004 (OR = 5.266, 95% CI: 2.241–12.378), 2006 (OR = 7.306, 95% CI: 2.925–18.250), 2009 (OR = 4.251, 95% CI: 1.431–12.627), and 2011 (OR = 37.882, 95% CI: 8.539–168.065). Among adolescents aged 15–18 years, older participants were less inclined to smoke in 2004 (OR = 0.531, 95% CI: 0.343–0.821) (Table 5).

### 3.6. Penalized Logistic Regression for Alcohol Consumption among Participants Aged 15–18 

Male adolescents were more likely to drink in 2004, 2006 and 2009. Among adolescents aged 15–18-year-old, older adolescents were less inclined to drink in 2004 (OR = 0.719, 95% CI: 0.527–0.980) and 2006 (OR = 0.716, 95% CI: 0.527–0.972). Participants who were not in school were more likely to drink in 2004 and 2009. Participants with previous smoking experience were more inclined to drink in 2004 (OR = 4.866, 95% CI: 2.050–11.546), 2006 (OR = 7.158, 95% CI: 2.851–17.974), 2009 (OR = 4.228, 95% CI: 1.374–13.013), and 2011 (OR = 25.700, 95% CI: 6.541–100.972) (Table 5).

We got similar results about tobacco consumption in Table 3 and Table 5. The gender, current education status, and alcohol consumption were associated with the tobacco consumption among adolescents. We also got the similar results about alcohol consumption in Table 4 and Table 5. The gender, current education status, and tobacco consumption were associated with the alcohol consumption among adolescents. For the age, adolescents aged 15–16 years old were more inclined to smoke and drink. However, among adolescents aged 15–18 years old, older adolescents were less to smoke and drink.

## 4. Discussion

The current study identified adolescent high-risk groups for tobacco and alcohol consumption. Adolescents aged 15–16 years were more inclined to smoke and drink compared with those aged 12–14. However, among adolescents aged 15–18, older ones were more inclined to avoid smoking and drinking. Adolescents who were not in school were inclined to smoke. This study revealed that male adolescents who lived in urban areas were expected to consume alcohol. The tobacco and alcohol consumption among adolescents were related from 2004 to 2011. Thus, future health education and promotion measures to limit tobacco and alcohol consumption should consider sociodemographic factors such as gender, age group, residence, and current education status.

For tobacco consumption, this study determined that male adolescents who were not studying were expected to consume tobacco more than their peers who were female and studying. A global survey showed that male adolescents were more inclined to smoke [5]; this result is similar to our finding. Previous studies revealed that current education status was associated with tobacco consumption among adolescents [33,34]. We determined that adolescents who were not studying were inclined to smoke from 2004 to 2011. Previous research showed that family intervention based on brief physician intervention were effective in preventing smoking among adolescents [35]. Thus, family interventions should be considered for adolescents who were not in school. Web-based interventions can be effectively delivered to different populations [36], including adolescents who are highly receptive to Internet-based information. Therefore, more Internet-based interventions can be considered in the future. However, based on a survey in Jiangsu Province, China, many smokers were college students. Approximately 29% of a past study’s sample (49% males and 5% females) reported smoking in the past 30 days [37]. This finding indicated that smokers started smoking for the first time after high school and before college.

Previous studies revealed that age was associated with tobacco consumption among adolescents [38,39]. Adolescents aged 15–16 years were inclined to smoke compared with those aged 12–14 in 2006 and 2009. This result is similar to the previous findings [5]. However, among adolescents aged 15–18, older ones were less inclined to smoke in 2004. Those aged 18 are usually in their third year of high school. Competition in China’s college entrance examination is so great that students have to spend all their free time on studies, which reduces the time for entertainment and parties. Fewer parties reduce the chances of teen smoking. Adolescents aged 12–18 are in middle and high schools. Thus, various age groups should be considered when designing future interventions on tobacco consumption among adolescents, and such interventions in middle and high schools should be different. Future studies should also consider many influential factors for specific age groups.

We did not found that residence (urban/rural) was associated with smoking behavior. However, previous study has determined that residence (urban/rural) had an impact on smoking [19]. In China’s rural areas, teens may be able to obtain cigarettes easily despite laws that prevent selling cigarettes to minors. In rural areas, cigarette susceptibility is higher than in urban areas [19]. Thus, the government should strengthen supervision and ban the sale of cigarettes to young people. Printing graphic warnings on cigarette packets can also help teenagers understand the dangers of smoking. The Chinese government should push for graphic warnings on cigarette packets as soon as possible.

For alcohol consumption, the present study revealed that male adolescents living in urban areas were more inclined to consume alcohol. A previous study revealed that gender was associated with alcohol consumption among high school students [24] and that females were not inclined to engage in drinking from 2004 to 2009. Male students were more inclined to drink than females. Previous studies also revealed that male adolescents were inclined to drink similar to their fathers [40], and non-drinking fathers opposed underage drinking. Traditional Chinese culture has a significant effect on men’s drinking behavior, such as in cases of social and business practices [41]. This culture is important for males. The parents’ attitude toward drinking can influence the adolescents’ drinking behavior [40]. In addition, one’s network can influence the drinking culture among young people. Adolescents obtained considerable information on drinking and shared this experience with their peers [42]. This condition increased the risk of drinking among adolescents. This finding can be attributed to the fact that drinking behavior is often supported by one’s family. Chinese adults believe that drinking is important, especially among males. Thus, adults allow male teenagers to drink alcohol at family gatherings.

Meanwhile, age is significantly related to drinking in 2006 and 2011. Adolescents aged 15–16 years were more inclined to drink than those aged 12–14; however, in the 15–18 age cluster, older adolescents were less inclined to drink probably because of academic stress and less chances of partying, which reduces their exposure to alcohol [43]. A previous study revealed that drinking rates were higher for high school than middle school students [44]. This finding is similar to that of our study. We determined that residence (urban or rural) was associated with drinking behavior. For future drinking interventions, considerable attention should be paid to adolescents who are not in school and are living in urban areas. Moreover, the government should design different interventions for urban and rural teenagers. 

Among adolescents, tobacco and alcohol consumption patterns were related from 2004 to 2011. This finding is similar to previous results, which indicated that cigarette smoking was strongly associated with alcohol consumption in young adults [45,46]. A previous study also revealed that drinking increased the desire and pleasure associated with smoking [45]. Another study indicated that students who started smoking in college were at greater risk of increased alcohol consumption [47]. Adolescents’ drinking behavior mostly occurred during parties with classmates. Moreover, non-smokers easily became smokers at parties. In China, smoking is considered as a catalyst of friendship and social activities [38], and offering cigarettes to others is considered a form of etiquette regardless of whether others smoke or not. However, adult smokers rarely give cigarettes to teenagers because adolescents’ smoking behavior is generally considered a bad habit by adults. Adult smokers offer cigarettes to other adults, especially men. Adolescents copy this behavior by giving cigarettes to fellow adolescents [48]. Thus, adolescents should be taught to reject cigarettes given by peers.

Many studies have been conducted on the association between smoking and drinking among college students, but only a few have examined the link between these behaviors among middle and high school students. Future studies should thus focus on the relationship and factors between smoking and drinking among middle and high school students. The present study revealed that a high correlation existed between smoking and alcohol consumption, which indicated that students who drank were inclined to start smoking and those who smoked were inclined to start drinking. Thus, schools should pay considerable attention to students who either drink or smoke to prevent them from starting the other behavior. Attitude, knowledge [49], self-efficacy, and cues to action [50] are considered strong predictors of smoking behavior. Future studies should consider the predictive effect of one behavior on another. 

Although the present study focused on adolescents who smoke or drink alcohol, some non-smoking/non-drinking adolescents are in similar sociodemographic situations. Future research may be useful in studying why adolescents in similar circumstances do not smoke/drink alcohol. Factors that influence the avoidance of smoking or drinking may be a key intervention point to reduce the incidence of tobacco and alcohol consumption among adolescents.

Several limitations can be observed in our study. First, CHNS respondents in different years have changed, and such changes may have affected the smoking and drinking status of the survey participants. Second, CHNS is not a nationwide survey. It was conducted only in 12 cities in China; hence, it is not representative of the actual conditions throughout the country. Third, the 2014 data on CHNS were not made available to the public. Therefore, we were unable to investigate the smoking and drinking trends in 2014 as well as the changes in the sociodemographic factors in that year. Fourth, many social demographic factors related to tobacco and alcohol consumption were not included in the questionnaire. Finally, we cannot determine if adolescents reported their real smoking/drinking state because the CHNS takes the form of household surveys, and teenagers may give false answers to their parents. Despite these limitations, this study has advantages. First, the data used in this study are based on longitudinal research (CHNS). Second, the large sample size can effectively reflect the actual smoking and drinking status of adolescents. Third, this study focuses on the demographic factors that influence smoking/drinking among adolescents. Identifying groups of high-risk adolescents has implications for future intervention studies.

## 5. Conclusions

Several sociodemographic factors are associated with tobacco and alcohol consumption from 2004 to 2011. The prevalence of tobacco and alcohol consumption among adolescents (12–18 years old) has not changed significantly. The current study identified adolescent high-risk groups for tobacco and alcohol consumption. The findings provide an improved understanding of the sociodemographic factors that influence tobacco and alcohol consumption among adolescents in China. We recommend that future intervention programs focus on these sociodemographic factors.

## Figures and Tables

**Table 1:** Characteristics of 3176 study participants stratified by survey year (N, %), CHNS.

Variable		2004	2006	2009	2011	Total	Smoker ^3^	Drinker ^4^
		*N* (%)	*N* (%)	*N* (%)	*N* (%)	*N* (%)	*N* (%)	*N* (%)
Gender	Male	580 (53.8)	406 (54.7)	331 (54.1)	380 (51.7)	1697 (53.6)	107 (99.1)	192 (81.4)
	Female	498 (46.2)	336 (45.3)	281 (45.9)	355 (48.3)	1470 (46.4)	1 (0.9%)	44 (18.6)
Ethnic group	Han	957 (88.8)	643 (86.7)	523 (85.5)	653 (88.8)	2776 (87.7)	99 (91.7)	219 (92.8)
	Minority	121 (11.2)	99 (13.3)	89 (14.5)	82 (11.2)	391 (12.3)	9 (8.3)	17 (7.2)
Residence	Urban	346 (32.1)	252 (34.0)	189 (30.9)	310 (42.2)	1097 (34.6)	44 (40.7)	106 (44.9)
	Rural	732 (67.9)	490 (66.0)	423 (69.1)	425 (57.8)	2070 (65.4)	64 (59.3)	130 (55.1)
Age	12–14 years old	485 (45.8)	331 (44.6)	353 (57.7)	387 (52.7)	1556 (49.1)	6 (5.6)	47 (19.9)
	15–16 years old	347 (32.2)	216 (29.1)	156 (25.5)	199 (27.1)	918 (29.0)	27 (25.0)	70 (29.7)
	17 years old	127 (11.8)	100 (13.5)	49 (8.0)	79 (10.7)	355 (11.2)	34 (31.5)	54 (22.9)
	18 years old	119 (11.0)	95 (12.8)	54 (8.8)	70 (9.5)	338 (10.7)	41 (38.0)	65 (27.5)
Current education status	Yes ^1^	879 (81.5)	603 (81.3)	527 (86.1)	664 (90.3)	2673 (84.4)	25 (23.1)	128 (54.2)
	No ^2^	199 (18.5)	139 (18.7)	85 (13.9)	71 (9.7)	494 (15.6)	83 (76.9)	108 (45.8)
Household registration type	Urban	386 (35.8)	284 (38.3)	228 (37.3)	364 (49.5)	1262 (39.8)	28 (25.9)	72 (30.5)
	Rural	692 (64.2)	458 (61.7)	384 (62.7)	371 (50.5)	1905 (60.2)	80 (74.1)	164 (69.5)
Father lives with family/adolescent child	Yes	933 (86.5)	642 (86.5)	526 (85.9)	615 (83.7)	2716 (85.8)	95 (88.0)	211 (89.4)
	No	145 (13.5)	100 (13.5)	86 (14.1)	120 (16.3)	451 (14.2)	13 (12.0)	25 (10.6)
Mother lives with family/adolescent child	Yes	1005 (93.2)	681 (91.8)	535 (87.4)	640 (87.1)	2861 (90.3)	102 (94.4)	222 (94.1)
	No	73 (6.8)	61 (8.2)	77 (12.6)	95 (12.9)	306 (9.7)	6 (5.6)	14 (5.9)

^1^ School pupils; ^2^ Participants who do not attend school; ^3^ Participants who had previous smoking experience; ^4^ Participants who had previous smoking experience.

**Table 2:** Trends in prevalence of alcohol and tobacco consumption among Chinese adolescents (12–18 years old) by survey year, CHNS.

Variable	2004	2006	2009	2011	Pearson Chi-Square	*p*-Value
	*N* **^5^**	*N*	*N*	*N*		
Smoking ^1^	33	33	23	19	X^2^ = 4.566	0.206
Non-smoking ^2^	1045	709	589	716		
%	3.06%	4.45%	3.76%	2.59%		
Drinking ^3^	65	59	52	60	X^2^ = 4.938	0.176
Non-drinking ^4^	1013	683	560	675		
%	6.03%	7.95%	8.50%	8.16%		

^1^ Participants who had previous smoking experience; ^2^ Participants who had no previous smoking experience; ^3^ Participants who had previous drinking experience; ^4^ Participants who had no previous drinking experience; ^5^ Number of participants.

**Table 3:** Penalized logistic regression analysis for tobacco consumption among Chinese adolescents (from 12–18 years old), CHNS.

		Tobacco Consumption											
Variable		2004			2006			2009			2011		
		Estimate	SE ^1^	*p*-Value	Estimate	SE	*p*-Value	Estimate	SE	*p*-Value	Estimate	SE	*p*-Value
Gender	Male												
	Female	−1.8592	0.6621	0.0050	−1.8779	0.6468	0.0037 *	−1.6728	0.6546	0.0106 *	−0.8176	0.3952	0.0386 *
Ethnic group	Han												
	Minority	−0.0529	0.3656	0.8849	0.2997	0.3159	0.3428	−0.0152	0.3978	0.9694	−0.6627	0.7576	0.3817
Residence	Urban												
	Rural	−0.3159	0.2377	0.1838	−0.4170	0.2393	0.0814	−0.3380	0.2981	0.2568	0.4432	0.3191	0.1649
Age	12–14 years old												
	15–16 years old	0.7850	0.5160	0.1282	1.4008	0.6458	0.0301 *	1.4825	0.6525	0.0231 *	0.1093	0.5991	0.8552
	17 years old	0.5900	0.3600	0.1013	−0.3835	0.3837	0.3175	0.0775	0.4014	0.8468	0.2369	0.4756	0.6184
	18 years old	−0.9331	0.3297	0.0047 *	−0.4424	0.3993	0.2680	−0.6444	0.4539	0.1557	0.2158	0.5817	0.7107
Current education status	Yes												
	No	0.9538	0.2440	<0.0001 **	0.9922	0.2432	<0.0001 **	0.7500	0.3185	0.0185 *	1.4647	0.3606	<0.0001 **
Household registration type	Urban												
	Rural	0.2549	0.2599	0.3266	0.2573	0.4248	0.2573	0.2545	0.3156	0.4200	−0.6840	0.3681	0.0631
Father lives in the household	Yes												
	No	−0.0238	0.3376	0.9439	0.3344	0.0313	0.3344	0.1710	0.4721	0.7172	0.0165	0.3738	0.9647
Mother lives in the household	Yes												
	No	−0.0110	0.4845	0.9819	0.1209	0.4840	0.8027	0.2445	0.4321	0.5715	0.4891	0.4080	0.2306
Alcohol consumption	Non-drinker												
	Drinker	0.8095	0.2164	0.0002 **	1.0622	0.2306	<0.0001 **	0.8531	0.2689	0.0015 *	1.6777	0.3312	<0.0001 **

^1^ SE, standard error, * Statistically significant (*p* < 0.05), ** Statistically significant (*p* < 0.05).

**Table 4:** Penalized logistic regression analysis for alcohol consumption among Chinese adolescents (from 12–18 years old), CHNS.

		Alcohol Consumption											
Variable		2004			2006			2009			2011		
		Estimate	SE	*p*-Value	Estimate	SE	*p*-Value	Estimate	SE	*p*-Value	Estimate	SE	*p*-Value
Gender	Male												
	Female	−0.9399	0.2119	<0.0001 **	−0.5756	0.1870	0.0021 *	−0.4184	0.1862	0.0247 *	−0.2702	0.1545	0.0804
Ethnic group	Han												
	Minority	−0.0866	0.2517	0.7308	−0.2890	0.2728	0.2894	−0.2341	0.2834	0.4088	−0.1057	0.2601	0.6844
Residence	Urban												
	Rural	−0.3931	0.1645	0.0169 *	−0.3932	0.1625	0.0155 *	−0.3289	0.1797	0.0673	−0.1899	0.1601	0.2355
Age	12–14 years old												
	15–16 years old	0.4001	0.2841	0.1592	1.0915	0.3194	0.0006 **	0.0982	0.3092	0.7508	1.1900	0.2769	<0.0001 **
	17 years old	0.2352	0.2303	0.3072	0.0800	0.2485	0.7475	0.3495	0.2899	0.2280	−0.2568	0.2336	0.2717
	18 years old	0.0609	0.2842	0.8303	−0.3494	0.2718	0.1986	−0.3167	0.3520	0.3684	−0.8783	0.2861	0.0021 *
Current education status	Yes												
	No	0.7603	0.1721	<0.0001 **	0.0204	0.1884	0.9136	0.7482	0.2174	0.0006 **	−0.0620	0.2298	0.7872
Household registration type	Urban												
	Rural	0.1140	0.1744	0.5133	0.2868	0.1691	0.0898	0.1215	0.1890	0.5203	0.6177	0.1721	0.0003 **
Father lives in the household	Yes												
	No	0.2293	0.2160	0.2883	−0.4381	0.3345	0.1903	−0.4024	0.3505	0.2510	0.1681	0.2075	0.4177
Mother lives in the household	Yes												
	No	−0.6880	0.4576	0.1327	−0.3152	0.4539	0.4875	0.1722	0.2741	0.5298	0.0330	0.2401	0.8907
Tobacco consumption	Non-smoking												
	Smoking	0.8049	0.2183	0.0002 **	1.0241	0.2329	<0.0001 **	1.0244	0.2789	0.0002 **	1.5298	0.3157	<0.0001 **

* Statistically significant (*p* < 0.05), ** Statistically significant (*p* < 0.05).

**Table 5:** Penalized logistic regression for tobacco consumption and alcohol consumption among Chinese adolescents (from 15–18 years old), CHNS ^a^.

		Tobacco Consumption				Alcohol Consumption			
Survey Year		2004	2006	2009	2011	2004	2006	2009	2011
Parameter		OR (95% CI)	OR (95% CI)	OR (95% CI)	OR (95% CI)	OR (95% CI)	OR (95% CI)	OR (95% CI)	OR (95% CI)
Gender	Male	1.000	1.000	1.000	1.000	1.000	1.000	1.000	1.000
	Female	***0.029 (0.002–0.395)***	***0.024 (0.002–0.313)***	***0.041 (0.003–0.575)***	0.213 (0.041–1.115)	***0.110 (0.037–0.329)***	***0.326 (0.148–0.719)***	*0.098 (0.025–0.388)*	0.592 (0.291–1.205)
Ethnic group	Han	1.000	1.000	1.000	1.000	1.000	1.000	1.000	1.000
	Minority	0.925 (0.211–4.061)	2.025 (0.560–7.326)	1.064 (0.207–5.467)	0.148 (0.004–5.587)	0.918 (0.297–2.835)	0.726 (0.237–2.226)	0.720 (0.170–3.043)	0.882 (0.269–2.890)
Residence	Urban	1.000	1.000	1.000	1.000	1.000	1.000	1.000	1.000
	Rural	0.617 (0.227–1.676)	0.487 (0.188–1.264)	0.525 (0.162–1.705)	2.351 (0.624–8.858)	0.557 (0.265–1.170)	***0.423 (0.212–0.841)***	0.414 (0.153–1.121)	0.508 (0.244–1.057)
Age	—	***0.531 (0.343–0.821)***	0.950 (0.627–1.438)	0.803 (0.494–1.304)	0.714 (0.397–1.282)	***0.719 (0.527–0.980)***	***0.716 (0.527–0.972)***	0.809 (0.543–1.204)	1.005 (0.730–1.383)
Current education status	Yes	1.000	1.000	1.000	1.000	1.000	1.000	1.000	1.000
	No	***5.197 (1.922–14.058)***	***7.349 (2.775–19.458)***	***5.161 (1.385–19.235)***	***9.286 (2.108–40.901)***	***3.941 (1.912–8.123)***	1.003 (0.470–2.142)	***6.335 (2.136–18.790)***	***1.019 (0.394–2.639)***
Household registration type	Urban	1.000	1.000	1.000	1.000	1.000	1.000	1.000	1.000
	Rural	1.627 (0.553–4.784)	1.381 (0.505–3.780)	1.218 (0.337–4.399)	0.201 (0.036–1.120)	1.058 (0.491–2.283)	1.634 (0.806–3.315)	1.052 (0.354–3.126)	3.378 (1.568–7.278)
Father lives in the household	Yes	1.000	1.000	1.000	1.000	1.000	1.000	1.000	1.000
	No	0.826 (0.201–3.393)	2.906 (0.698–12.109)	0.478 (0.031–7.375)	2.145 (0.419–10.967)	1.355 (0.501–3.667)	0.316 (0.066–1.504)	0.770 (0.120–4.943)	1.342 (0.496–3.632)
Mother lives in the household	Yes	1.000	1.000	1.000	1.000	1.000	1.000	1.000	1.000
	No	1.236 (0.174–8.785)	1.632 (0.228–11.669)	1.116 (0.134–9.257)	1.081 (0.147–7.955)	0.336 (0.052–2.172)	0.706 (0.111–4.506)	0.994 (0.169–5.845)	1.332 (0.424–4.185)
Alcohol consumption	Non-smoking	1.000	1.000	1.000	1.000	—	—	—	—
	Smoking	***5.266 (2.241–12.378)***	***7.306 (2.925–18.250)***	***4.251 (1.431–12.627)***	***37.882 (8.539–168.065)***	—	—	—	—
Tobacco consumption	Non-drinker	—	—	—	—	1.000	1.000	1.000	1.000
	drinker	—	—	—	—	***4.866 (2.050–11.546)***	***7.158 (2.851–17.974)***	***4.228 (1.374–13.013)***	***25.700 (6.541–100.972)***

^a^ The regression model using age as a continuous variable and excluding those adolescents aged below 14 years old.
